# The Effect of Loneliness on Cognition: A Longitudinal Study

**DOI:** 10.1093/geroni/igaa057.1032

**Published:** 2020-12-16

**Authors:** Robert Maiden, Larry Greil, Bert Hayslip

**Affiliations:** 1 Alfred University, Alfred, New York, United States; 2 Sociaology, Alfred, United States; 3 University of North Texas, Denton, Texas, United States

## Abstract

A panel study was conducted among 244 older adults (52-years-old to 92) to explore whether social engagement and loneliness are associated with cognitive ability. Measures of crystallized (Gc) and fluid (Gf) ability were collected at two points in time. Using latent variable SEM with separate models for men and women, Gc and Gf at W2 were regressed on perceived general health, social support, sociability, loneliness and involvement in organizational activities, controlling for Gc and Gf at W1. Fit statistics were adequate. Among women, Gc at W1 was associated with perceived health (B=1.03, p=.000), while Gf at W1 was associated with perceived general health (B=1.28, p=.010) and organizational involvement (B=1.8, p=.019). Gc at W2 was associated with Gc at W1 (B=.61, p=.000), and age (B=-.12, p=.007), while Gf at W2 was associated with Gf at W1 (B=.74, p=.000), age (B=-.08, p=.008), and loneliness (B=-.78, p=.038). Among men, there were no significant associations between either Gc at W1 or Gf at W1 and other variables. Gc at W2 was associated with Gc at W1 (B=.29, p=.031), while Gf at W1 was associated with Gf at W2 (B=7.9, p=.000) and perceived general health (B=2.46, p=.006). These findings suggest that loneliness and organizational involvement are associated with lower Gf scores among women but not among men. Gc was not associated with loneliness or organizational involvement for either women or men. This suggests that interventions targeting the prevention of loneliness and the promotion of organizational involvement may enhance cognitive functioning in later life among women.

